# The Role of Secretory Autophagy in Zika Virus Transfer through the Placental Barrier

**DOI:** 10.3389/fcimb.2016.00206

**Published:** 2017-01-09

**Authors:** Zhong-Wei Zhang, Zi-Lin Li, Shu Yuan

**Affiliations:** ^1^College of Resources, Sichuan Agricultural UniversityChengdu, China; ^2^General Hospital of Lanzhou Military RegionLanzhou, China

**Keywords:** Zika virus, microcephaly, secretory autophagy, exosome, placental barrier

## Abstract

Recent studies indicated that the Zika virus genome could be detected in the amniotic fluid and the fetal brain, which confirms that the virus can cross the placental barrier. Secretory autophagy or exosome pathways may participate in this virus transfer. Autophagy modulators regulate autophagosome formation or membrane fusion with lysosomal vesicles and therefore inhibit viral nucleocapsid releasing or virus transfer to the fetus hypothetically. However, some autophagy modulators may enhance virus replication. Autophagy inhibitors may arrest placental development; while exaggeration of autophagy in human placenta may be associated with the fetal growth restriction. Therefore, autophagy modulators should be used carefully due to their complex clinical effects. Alternatively, exosome-specific inhibitors might be also considered, although their safety of both maternal and fetal conditions must be carefully assessed before any advancement to human clinical trials.

## Introduction

A widespread epidemic of Zika virus (an emerging mosquito-borne flavivirus) infections was found in Central and South America recently. The main concern is the significant increasing of microcephaly in the fetus born to the mother of Zika virus infection (Faria et al., 2016). Placenta may play an important role in the virus transfer. One possibility is that the Zika virus can penetrate through the placental barrier. Alternatively, the infection to the placenta might induce some immune responses and cause brain defects indirectly (Adibi et al., 2016a). A recent study indicated that the viral genome could be detected in the amniotic fluid, which confirms that the virus could penetration through the placental barrier (Calvet et al., 2016). The complete genome of Zika virus can be also recovered from the fetal brain, which confirms the tropism of Zika virus for neural tissues (Mlakar et al., 2016). Direct evidences of fluorescence in situ hybridization (FISH) confirmed the presence of Zika virus RNA in mutiple mouse trophoblast cells, such as glycogen trophoblasts, mononuclear trophoblasts, spongio-trophoblasts, and syncytio-trophoblasts. Transmission electron microscopy of the mouse placenta found many 50-nm bodies in the endoplasmic reticulum of trophoblast cells (Miner et al., 2016). Indirect immunoblotting with the serum obtained from the infected mother found fluorescence signals in the destroyed neuron cells. And negative staining of the infected fetal brain tissue showed 42–54 nm spherical virus particles (Mlakar et al., 2016). Therefore the hypothesis that the virus could penetration through the placental barrier has been confirmed.

## Secretory autophagy may mediate Zika virus transfer

**Figure 1:**
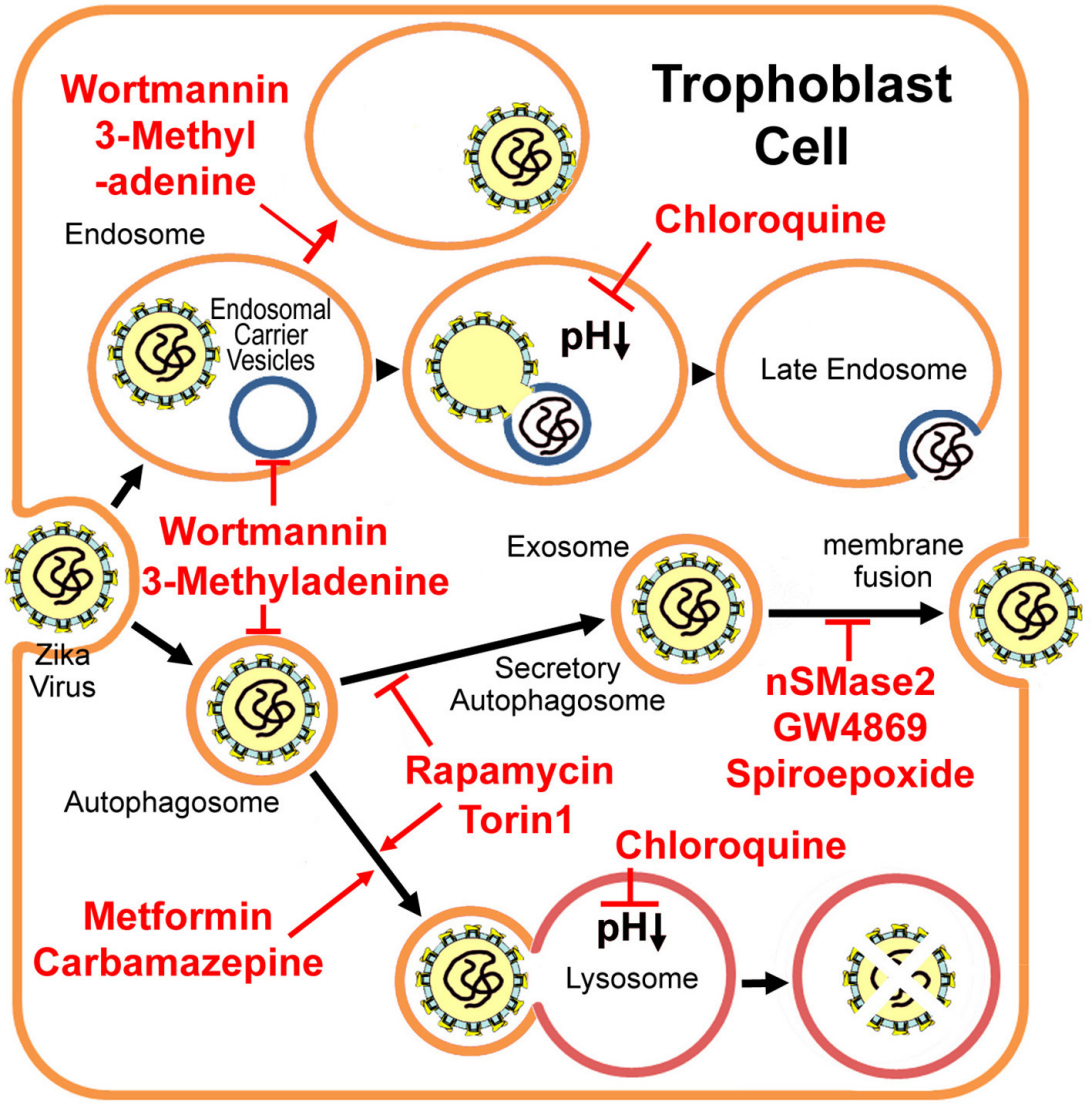
**Putative Zika virus entry pathway and the secretory autophagy/exosome pathway in the trophoblast cell**. 3-methyladenine and wortmannin inhibit both autophagosome formation and endosomal carrier vesicle formation (Nour et al., 2013). In the absence of these vesicles, Zika virus may either fail to fuse completely with the early endosome membrane or it may fuse with as yet unidentified endosomal compartments in which the nucleocapsid remains trapped (Nour et al., 2013). Chloroquine and its derivative hydroxychloroquine are lysosomal lumen alkalizers, which inhibit degradative autophagy by neutralizing the acidic pH in the lumen of lysosomal vesicles (Vakifahmetoglu-Norberg et al., 2015). Metformin (Fedson, 2013) and Carbamazepine (Schiebler et al., 2015) induce autophagic killing of pathogenic microbes (degradative autophagy). mTORC inhibitors Torin 1 and rapamycin stimulate autophagy, but inhibit exosome release evidently (Fader et al., 2008), therefore limiting Zika virus transfer through the placental barrier. However, Torin 1 and rapamycin may enhance virus replication (Hamel et al., 2015). Alternatively, exosome-specific inhibitors nSMase2, GW4869 and spiroepoxide (Li et al., 2013) may prevent the transfer, although their drug safeties need further studies.

However, we still do not know by what exact mechanism Zika virus transfers through the trophoblast cell. Like dengue virus, Zika virus may be packaged as a cargo for the placental exosome pathway at the trophoblast endoplasmic reticulum, which is closely associated with the “secretory autophagy” process (Chahar et al., 2015; Carneiro and Travassos, 2016). Autophagy usually has a protective role by removing damaged organelles and protein aggregates and maintaining cellular homeostasis (Ponpuak et al., 2015). Contrasting to the degradative autophagy, there is also a non-degradative autophagic machinery, termed secretory autophagy (Ponpuak et al., 2015). Some proteins lack the N-terminal leader peptides, and therefore cannot enter the common endoplasmic-reticulum-to-Golgi-apparatus secretory pathway. And these proteins (such as viral capsid polypeptides) may be exported through the secretory autophagy pathway unconventionally (Ponpuak et al., 2015). Different from the degradative autophagy (fusion with the lysosome), the secretory autophagy pathway may cause expulsion or secretion of the viral particle other than its degradation (Figure 1). Zika virus infection is associated with the observation of a lot of double membrane intracytoplasmic vacuoles (namely the autophagosome). While the co-detection of the virus envelope peptides and the autophagy marker protein LC3 (cytosolic microtubule-associated light chain 3) has been reported (Hamel et al., 2015). Therefore, secretory autophagy (or exosome) may facilitate Zika virus transfer across the placental barrier, and regulations to the equilibrium between degradative autophagy and secretory autophagy may influence the incidence of microcephaly.

## Autophagy inhibitors and the clinical effects

For some flaviviruses, upon binding to the receptor, the virus enters the early endosome via the clathrin-mediated endocytic process (Nour et al., 2013; Carneiro and Travassos, 2016). About 5 min later, the virus particle fuses with the endosomal carrier vesicle (ECV) membrane predominantly (Figure 1). However for several minutes, viral nucleocapsids remain trapped in the ECV lumen, until ECVs fuse back with the late endosome limiting-membrane. These fusion events require microtubule movement, some membrane fusion factors and the PI(3)P-mediated signaling (Nour et al., 2013). PI(3)P kinase inhibitors, 3-methyladenine and wortmannin, inhibit both autophagosome formation, and ECV formation (Nour et al., 2013). In the absence of ECVs, the virus either fails to fuse fully with the early endosome (only partial fusion to the proximal membrane) or it fuses with some other endosomal vesicles but the nucleocapsid still is trapped (Figure 1; Nour et al., 2013). Autophagosomes were observed in human skin fibroblast cells infected with Zika virus (Hamel et al., 2015). The specific autophagy inhibitor 3-methyladenine strongly reduces Zika viral copy numbers in the infected fibroblast cells (Figure 1 and Table 1; Hamel et al., 2015). However, neither 3-methyladenine nor wortmannin is an FDA (the Food and Drug Administration) -approved drug.

**Table 1:** **List of autophagy and exosome modulators and the clinical effects to virus or bacterial infections**.

**Drug's name**	**Pathways involved in**	**Clinical effects**
3-Methyladenine Wortmannin	PI(3)P kinase inhibitors; Autophagy inhibitors	Reduce Zika viral copy numbers in the infected fibroblast cells (Hamel et al., 2015)
Chloroquine Hydroxychloroquine	Lysosomal lumen alkalizers; Autophagy inhibitors	Decrease Dengue virus type 2 replication in Aotus monkeys (Farias et al., 2015)
Metformin	AMPK activator; Autophagy stimulator	Enhances antigen processing and cytotoxic T lymphocytes after influenza virus infections (Fedson, 2013)
Carbamazepine	Induce inositol depletion -dependent autophagy	Kill intracellular *Mycobacterium tuberculosis*; reduce tuberculosis (Schiebler et al., 2015)
Statin	Enhancing autophagy and phagosome maturation	Reduces the *Mycobacterium tuberculosis* burden in human macrophages (Parihar et al., 2014)
Apro-autophagic peptide Tat-beclin 1	Induce endoplasmic reticulum stress-associated autophagy	Protect against neuronal cell death induced by the West Nile virus infection (Shoji-Kawata et al., 2013)
Torin 1 Rapamycin	mTORC1 inhibitor; Autophagy stimulator	May prevent Zika virus transfer through the placental barrier (Fader et al., 2008), but increase virus replication (Hamel et al., 2015)
nSMase2 GW4869 spiroepoxide	Neutral sphingomyelinase inhibitor; Exosome inhibitor	May prevent Zika virus transfer through the placental barrier (Li et al., 2013)

The cellular alkalizers also repress degradative autophagy through neutralizing lysosomal acidic pH, which is necessary for lysosomal hydrolases activations (Vakifahmetoglu-Norberg et al., 2015). Chloroquine and its derivative hydroxychloroquine are such alkalizers and are used clinically as anticancer drugs or antimalarial candidate medicines (Vakifahmetoglu-Norberg et al., 2015). Chloroquine had inhibitory effects on flavivirus replication *in vitro* and significantly decreased Dengue virus type 2 replication in Aotus monkeys (Farias et al., 2015), but it did not reduce the duration of viral infection in a human clinical trial and showed several adverse effects, primarily vomiting (Tricou et al., 2010). More importantly, chloroquine seems not to be related with the equilibrium between degradative autophagy and secretory autophagy (Figure 1 and Table 1).

In general, viruses customize autophagy protein for efficient viral entry (Pirooz et al., 2014). However, some opposite reports suggest that the involvement of autophagy in flavivirus infection is controversial. ATG16L2 (Autophagy related 16-like 2) was identified among the top 30 genes down-regulated in human neural stem cells infected with Zika virus (Rolfe et al., 2016). *LC3* transcript was also down-regulated by Zika virus infection in mice brain cells (Li et al., 2016). However, West Nile virus infection did not induce LC3 lipidation in multiple mammalian cell lines (Vandergaast and Fredericksen, 2012). And depletion of autophagy-related (ATG) protein ATG5 does not affect replication of West Nile virus (Vandergaast and Fredericksen, 2012; Martín-Acebes et al., 2015). For mouse embryonic fibroblast cells infected with Japanese encephalitis virus, either depletion in ATG7 or deficiency in ATG5 would result in higher viral replication levels (Sharma et al., 2014). Autophagy may play a positive role in the early infection stages, however it becomes dysfunctional when the misfolded proteins accumulate at the late stages. Autophagy-deficient cells were highly susceptible to virus-induced cell death (Sharma et al., 2014). Therefore, the role of autophagy may be varied at different infection stages. Autophagy inhibitors should be used carefully because of their complex clinical effects.

The autophagy markers LC3-II and Beclin-1 are highly expressed in villous cytotrophoblasts at the first trimester and they are prevalently activated in trophoblast cells during the whole gestation period (Chifenti et al., 2013). The cytotrophoblast and the syncytiotrophoblast, which form the placental barrier, function differently according to different gestational ages. Autophagy plays an important role in the placental development and pregnancy maintaining (Gong and Kim, 2014). Therefore, autophagy inhibitors may produce adverse effects to the fetus.

## Autophagy stimulators and the clinical effects

Metformin, a type 2 diabetes drug, induces autophagy by activating AMPK (adenosine monophosphate-activated kinase) pathways, may enhance antigen presentation and processing and improve maintenance of memory CD8 cytotoxic T lymphocytes after influenza virus infections (Fedson, 2013). Carbamazepine, an anticonvulsant drug, was indicated to trigger inositol-depletion dependent degradative autophagy of intracellular Mycobacterium tuberculosis (a pathogenic bacteria) in macrophages, which relieves pulmonary symptoms and activate mouse's immunity against the bacterium (Schiebler et al., 2015). Statin (a lipid-lowering medicine) reduced the M. tuberculosis proliferation in human and mouse macrophage cells through promoting phagosome maturation (Parihar et al., 2014). Membrane remodeling and viral replication usually induce the endoplasmic-reticulum stresses that cause the unfolded-protein responses (Blázquez et al., 2014). Concomitant with the induction of the unfolded protein response, flavivirus infections induced autophagy-related pathway activation has been also described (Blázquez et al., 2014). For the instance of West Nile virus, stimulation of autophagy via apro-autophagic peptide (Tat-beclin 1-derived from a region of the autophagy protein beclin 1) can protect neuronal cells from cell death caused by the virus infection (Shoji-Kawata et al., 2013). Thus, drugs that promote autophagy might have broad therapeutic applications (Table 1; Zumla et al., 2016).

Among the common autophagy stimulators, only rapamycin (a mTORC1 inhibitor) has been shown to inhibit exosome release evidently (Fader et al., 2008). Given that exosome may play a key role in Zika virus transfer, rapamycin might limit Zika virus transfer through the placental barrier. However, Torin 1 (another mTORC inhibitor) greatly enhances Zika virus replication (Hamel et al., 2015). Thus, the rapamycin may not be used as an efficient drug for the treatment of Zika virus infection.

Furthermore, misregulated autophagy may result in adverse consequences to the fetus. For example, exaggeration of autophagy in human placenta is associated with the fetal growth restriction (Curtis et al., 2013). Therefore, autophagy stimulators should also be chosen carefully due to their complex clinical effects.

## Exosome-specific inhibitors

Until now, the roles of autophagy in replication and secretion have not been well-established as contradictory results have been reported for different viruses. Hereby, some drugs other than autophagy modulators should also be considered, such as exosome-specific inhibitors nSMase2, GW4869, and spiroepoxide (neutral sphingomyelinase inhibitors; Table 1; Li et al., 2013). Sphingomyelinase and related ceramide play important roles in embryo development and apoptosis (de Castro e Paula and Hansen, 2008). However, alkaline sphingomyelinase and neutral sphingomyelinase are functionally similar. Neutral sphingomyelinase inhibitors might not cause significantly adverse consequences to the fetus, because of the complementation of alkaline sphingomyelinase. Nevertheless during pregnancy, when the number of available drugs is exceedingly limited and the bar for approval is extremely high, we must be very cautious when testing any potential therapeutic that could be used in human pregnancy. The safety of these exosome inhibitors must be carefully assessed (especially in non-human primates) before any advancement to human clinical trials. Alternatively, new exosome inhibitors should be developed, such as some natural compounds from soil actinomycetes (Dong et al., 2005). Then their safety at both maternal and fetal conditions requires further studies.

## Potential combinations with other drugs

We should keep in mind that the secretory autophagy (exosome) may be only one of the complex mechanisms of Zika virus penetration through the placental barrier. Zika virus infection triggers apoptosis and vascular damages in the placenta, which may increase the permeability of the placenta (Miner et al., 2016). Therefore, Zika virus might be vertically transmitted independent of the secretory autophagy (exosome) pathway. A combination of antiviral agents and exosome inhibitors might be required to achieve the desired effect. For example, both type I interferons and type III interferons are induced by Zika virus infections (Bayer et al., 2016; Quicke et al., 2016), and interferon-λ restricts Zika virus replication in human trophoblast cells (Bayer et al., 2016). Although, interferons and associated cytokines may generate indirect teratogenic and/or neurotoxic effects to the fetus (Adibi et al., 2016b; Mor, 2016), interferon-α does not apparently increase the risk of miscarriage, major malformation, preterm delivery, or stillbirth (Yazdani Brojeni et al., 2012). Moreover, interferon-α may show some protective effects against pregnancy loss in the case of essential thrombocythemia (Yazdani Brojeni et al., 2012).

When Zika virus has penetrated the placenta, it may damage the fetal brain directly or indirectly, because of its neurotropic property (Mlakar et al., 2016). This assumption implies that the virus exists in the fetal cerebral cortex at the early gestation stages. However, at that time, the fetus is fairly-well shielded from maternal circulation. Maternal blood flows into the placenta only after 10 weeks of gestation (Burton et al., 2001). Consistent with these structure changes, a tissue-level analysis in a case where the mother was infected at 7 weeks and miscarried at 12 weeks confirmed that the trophoblast was not infected by Zika virus at that period (Noronha et al., 2016). When Zika virus reaches trophoblast cells, non-neutralizing antibody and the virus may form an immuno-complex (Toth et al., 1994). And the exosome may be formed at that time, which may help the immuno-complex to transfer through the placental barrier. Nevertheless, the transfer is unlikely to be happened before 16 weeks of gestation (Saji et al., 1999; Fuchs and Ellinger, 2004). Therefore, autophagy-related drugs or exosome inhibitors may be not effective for the infected pregnant woman before 16 weeks. Instead, for these patients, interferons may help their immune system to clear the virus (Bayer et al., 2016; Quicke et al., 2016). Autophagy modulators or exosome inhibitors may be more useful to the medium-late stage pregnant women.

## Author contributions

ZZ contributed to the discussion of ideas and helped with the writing. ZL contributed to the discussion of ideas and the writing. SY conducted the literature search and drafted the manuscript.

### Conflict of interest statement

The authors declare that the research was conducted in the absence of any commercial or financial relationships that could be construed as a potential conflict of interest. The reviewer IEC and handling Editor declared their shared affiliation and the handling Editor states that the process nevertheless met the standards of a fair and objective review.
